# Quantitative spectrofluorometric assay detecting nuclear condensation and fragmentation in intact cells

**DOI:** 10.1038/s41598-021-91380-3

**Published:** 2021-06-07

**Authors:** Pavlina Majtnerova, Jan Capek, Filip Petira, Jiri Handl, Tomas Rousar

**Affiliations:** Department of Biological and Biochemical Sciences, Faculty of Chemical Technology, University of Pardubice, Studentska 573, 532 10 Pardubice, Czech Republic

**Keywords:** Biochemical assays, Cell death, Apoptosis

## Abstract

At present, nuclear condensation and fragmentation have been estimated also using Hoechst probes in fluorescence microscopy and flow cytometry. However, none of the methods used the Hoechst probes for quantitative spectrofluorometric assessment. Therefore, the aim of the present study was to develop a spectrofluorometric assay for detection of nuclear condensation and fragmentation in the intact cells. We used human hepatoma HepG2 and renal HK-2 cells cultured in 96-well plates treated with potent apoptotic inducers (i.e. cisplatin, staurosporine, camptothecin) for 6–48 h. Afterwards, the cells were incubated with Hoechst 33258 (2 µg/mL) and the increase of fluorescence after binding of the dye to DNA was measured. The developed spectrofluorometric assay was capable to detect nuclear changes caused by all tested apoptotic inducers. Then, we compared the outcomes of the spectrofluorometric assay with other methods detecting cell impairment and apoptosis (i.e. WST-1 and glutathione tests, TUNEL, DNA ladder, caspase activity, PARP-1 and JNKs expressions). We found that our developed spectrofluorometric assay provided results of the same sensitivity as the TUNEL assay but with the advantages of being fast processing, low-cost and a high throughput. Because nuclear condensation and fragmentation can be typical markers of cell death, especially in apoptosis, we suppose that the spectrofluorometric assay could become a routinely used method for characterizing cell death processes.

## Introduction

Apoptosis is a complex process including various morphological and biochemical cellular changes that can be used for apoptosis characterization. The morphological changes include cell shrinkage, pyknosis and karyorrhexis followed by DNA fragmentation in late stage of apoptosis^1,2^. Then, the cellular cytoskeleton is damaged leading to membrane blebbing and, finally, apoptotic bodies formation^1–3^.

Pyknosis is an irreversible process of nuclear condensation commonly associated with early stages of apoptosis and necrosis^4^. Pyknosis can be divided into nucleolytic and anucleolytic pyknosis^5^. Nucleolytic pyknosis mainly occurs during apoptosis. Chromatin is condensed into large clumps which can be packed into apoptotic bodies. In contrast, anucleolytic pyknosis predominantly occurs during necrosis when chromatin condenses into smaller, irregular clumps^4,6^.

Karyorrhexis occurs during late stage of apoptotic and necrotic processes when nucleus is fragmented and chromatin irregularly distributed into the cytoplasm, followed by formation of apoptotic bodies and karyolysis, respectively. Karyolysis is a complete enzymatic degradation of chromatin in dying cells^7^. Nuclear condensation and fragmentation have been used as markers for late stages of apoptosis^8^.

Caspases play crucial roles in apoptosis. Caspases 8, 9 and 10 initiate apoptotic cascade through activation of effector caspases 3, 6 and 7^9^. DNA fragmentation factor (DFF) has an essential role in DNA cleavage during the apoptotic process. Activated caspase 3 cleaves the DNA fragmentation factor, and activates its catalytic subunit (DFF40)^10^. Activated DFF40 cleaves double-stranded DNA with a preference for adenine and thymine (A/T) rich region in internucleosomal linker regions into approx. 180 bp fragments and multiples thereof^1,11^. This characteristic DNA cleavage pattern is called “DNA ladder” and can be used for detection of apoptotic DNA fragmentation^1,12,13^. In addition to DNA ladder, comet and Terminal deoxynUcleotidyl transferase Nick-End Labeling (TUNEL) assays belong among frequently used methods for apoptotic DNA fragmentation detection. DNA ladder assay uses the presence of the DNA ladder fragments pattern occurring during apoptosis detected after electrophoresis in agarose gel, while comet detects fragmented DNA in the gel. In the TUNEL technique, free -OH moiety in the double and single strand DNA breaks is labeled using modified deoxynucleotides analogues tagged with various markers allowing DNA strand breaks detection^14^. Chromatin condensation and DNA fragmentation can be also characterized using microscopic methods, especially fluorescence microscopy. Hoechst fluorescent probes originate from bisbenzimides, a family of lipophilic substances, that bind preferentially to a small groove of A/T rich DNA sequences called A-T regions^15^. At least three consecutive A-T base pairs are required for specific Hoechst dye binding leading to fluorescence increase. In addition, some papers reported that the nucleus of apoptotic cells can exhibit enhanced fluorescence after Hoechst binding^16–18^. Therefore, Hoechst probes have been frequently used for nucleus staining in fluorescence microscopy and flow cytometry^19–21^. At present, three Hoechst probes have been used in vitro: 33258^22^, 33342^23^, 34580^24^. Hoechst 33258 is a lipophilic and cell permeable probe^25–27^ most frequently used in fluorescence microscopy for qualitative detection of nuclear morphology changes, primarily for detecting cell shrinkage, chromatin condensation, nuclear fragmentation and apoptotic bodies formation in various cell lines^28–32^. Despite the unique Hoechst probe properties for nuclear changes detection, no scientific study, however, reported a quantitative spectrofluorometric method development. Thus, the aim of the present study was to develop a Hoechst 33258 dye spectrofluorometric assay for quantitative measurement of nuclear condensation and fragmentation in intact cells. Finally, we aimed to evaluate the obtained outcomes in comparison with other frequently used methods for cell damage (i.e. WST-1 and glutathione assays) and apoptosis (TUNEL, DNA ladder, etc.) detection.

## Results

### Optimization of the spectrofluorometric assay

**Figure 1:**
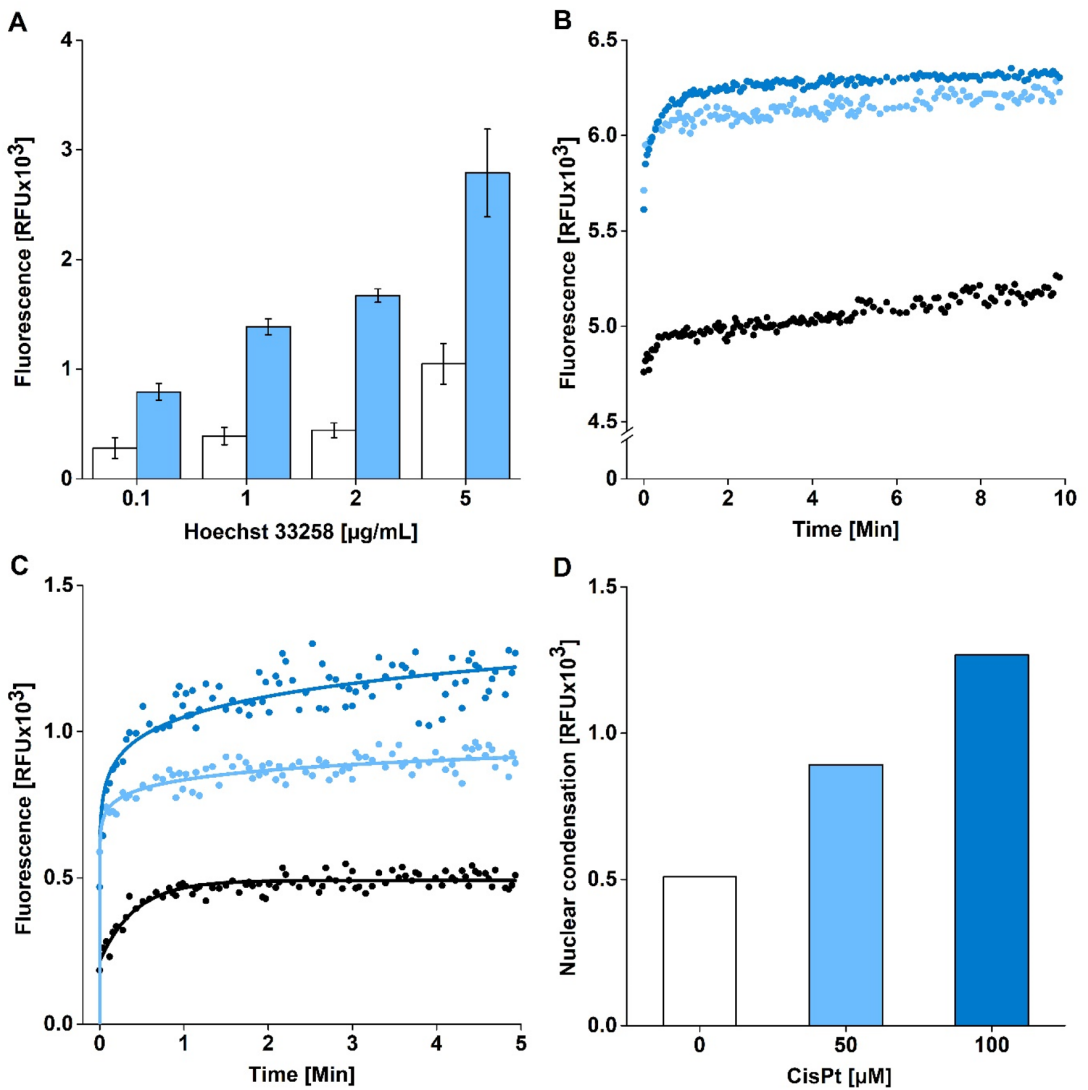
Optimization of the spectrofluorometric assay for detection of nuclear condensation and fragmentation in HepG2 cells. (**A**) Optimization of H33258 concentration (0.1; 1; 2; 5 µg/mL) – untreated cells (white columns), 100 µM cisplatin treated cells (blue columns). Mean ± SEM (n = 3). (**B**) Change of intensity of fluorescence over time. HepG2 cells were treated with CisPt for 24 h. Then, the cells were incubated with H33258 (2 µg/mL) and fluorescence (EX/EM = 352/461 nm) was recorded for 10 min. Untreated cells (black), 50 µM CisPt (light blue), 100 µM CisPt (dark blue). (**C**,**D**) Detection of nuclear condensation and fragmentation in cells. HepG2 cells were treated with cisplatin for 24 h. Then, the cells were incubated with H33258 (2 µg/mL) and fluorescence (EX/EM = 352/461 nm) was recorded for 5 min. Finally, the background fluorescence was subtracted and fluorescence intensity corresponding to the extent of nuclear condensation and fragmentation was showed in untreated (white), 50 µM (light blue) and 100 µM CisPt (dark blue) treated cells (= **C**). The fluorescence intensity attributed to the extent of nuclear condensation and fragmentation was compared among untreated cells (white), 50 µM CisPt (light blue), 100 µM CisPt (dark blue) treated cells (= **D**).

We aimed to develop a spectrofluorometric method for detection of nuclear condensation and fragmentation in the intact cells using a fluorescent probe Hoechst 33258. According to the literature, we used the same λ_(ex,max)_ = 352 nm and λ_(em,max)_ = 461 nm in H33258 measurements^33–35^, but optimized the experimental procedure, i.e. H33258 concentration and incubation length of the probe with cells. To induce cell damage, we incubated HepG2 cells with 100 µM cisplatin (CisPt) for 24 h. Then, we incubated the cells with the concentrations range of 0.1–5 µg/mL H33258 (Fig. 1A). The highest fluorescence signal was detected in 5 µg/mL H33258 in CisPt treated cells but the background fluorescence in untreated HepG2 cells was strongly enhanced too. Due to the highest signal-to-noise ratio found after the 2 µg/mL, we selected this H33258 treatment concentration to be optimal for following experiments.

For estimation of optimal cell incubation time with H33258, HepG2 cells were treated with CisPt (0; 50; 100 µM) for 24 h. We found that centrifugation of cells (5 min, 8000*g*; RT) after the treatment with tested compounds, followed by cell culture medium replacement, is crucial for achieving repeatable results because it ensures the sedimentation of all cells on the bottom of a well. After centrifugation, 70 µL of culture medium was replaced with 70 µL of PBS 1 × in each well. Then, 10 µL of H33258 was added to obtain final concentration 2 µg/mL H33258 in a well and fluorescence was recorded at EX/EM = 352/461 nm.

We found that fluorescence intensity was increasing strongly during the first minute of cells incubation with H33258 (Fig. 1B). Then, the fluorescence intensity (IF) remained rather stable between the second and the tenth minute of incubation implying that any of durations including this time interval could be used for the purposes of the spectrofluorometric assay. We selected 5 min of H33258 incubation with cells in all following experiments. In addition, we confirmed that IF was enhanced relatively to the increasing CisPt concentration in cells according to expected induction of nuclear condensation and fragmentation. Figure 1C shows IF detected in cells after subtraction of background fluorescence intensity in blank samples (i.e. without cells). Finally, the extent of nuclear condensation and fragmentation in cells was expressed in Relative Fluorescence Units (RFU) (Fig. 1D).

### Estimating sensitivity of the spectrofluorometric assay

**Figure 2:**
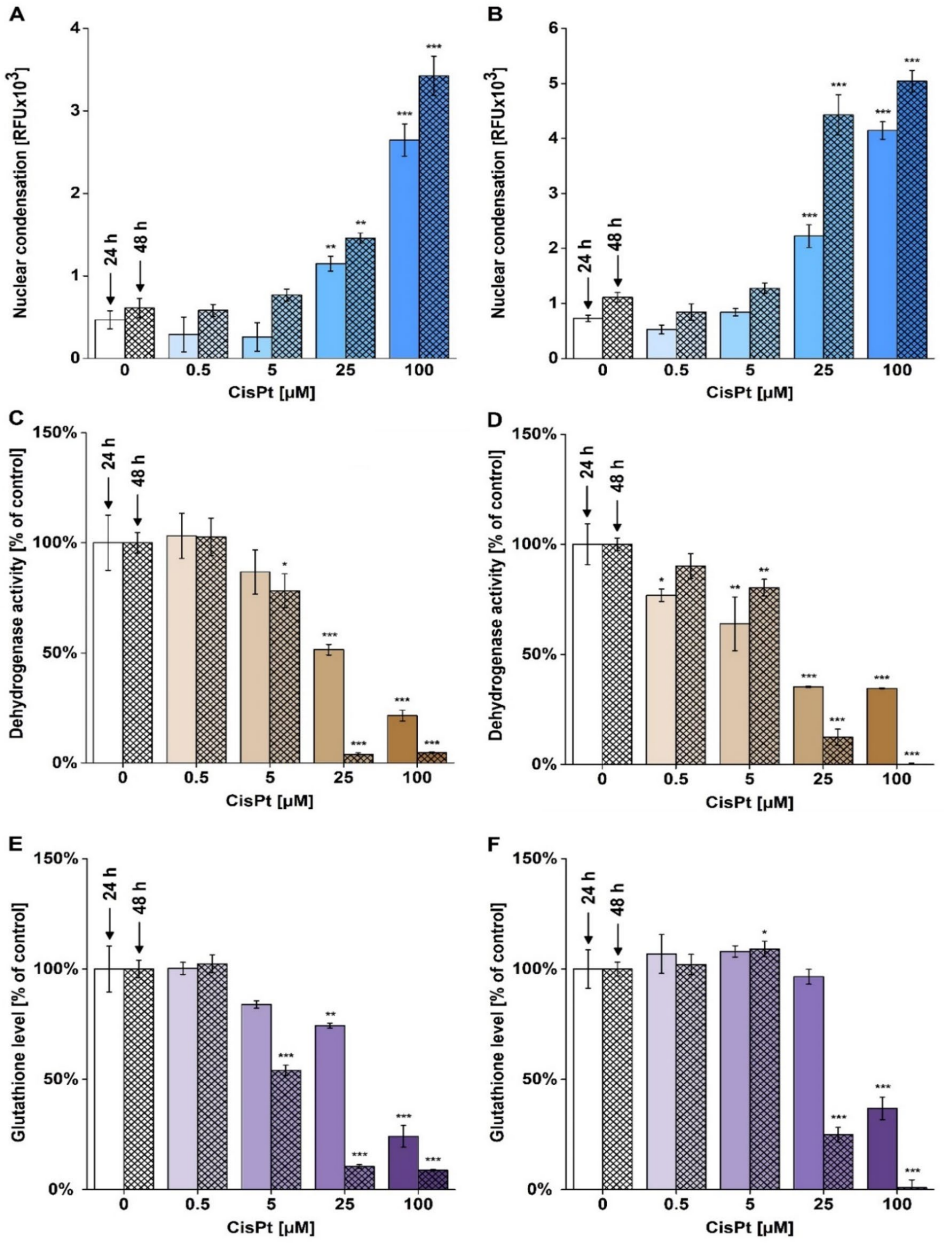
Characterizing cisplatin-induced toxicity in HepG2 and HK-2 cells. HepG2 (**A**,**C**,**E**) and HK-2 (**B**,**D**,**F**) cells were incubated with cisplatin (CisPt; 0; 0.5; 5; 25 and 100 µM) for 24 and 48 h. After treatment, nuclear condensation and fragmentation using the H33258 spectrofluorometric assay (**A**,**B**), dehydrogenase activity using the WST-1 test (**C**,**D**) and glutathione levels using monochlorobimane assay (**E**,**F**) were measured. The data are presented as mean ± SEM (A, B) and mean ± SD (C-F). (*p < 0.05; **p < 0.01; ***p < 0.001, vs. untreated cells at appropriate time interval).

Further aim of our study was to use the H33258 spectrofluorometric assay in cells exhibiting nuclear condensation and fragmentation of different origin and extent. We incubated HepG2 and HK-2 cells with CisPt (0; 0.5; 5; 25 and 100 µM) for 24 and 48 h. After H33258 treatment, we observed increasing IF relating to both CisPt doses and incubation time in HepG2 (Fig. 2A) and HK-2 cells (Fig. 2B). We found a significant increase of IF in 25 and 100 µM CisPt treated cells after 24 and 48 h. In addition, the extent of condensation and fragmentation detected after 24 and 48 h demonstrated time and dose dependent increase (Fig. 2A,B).

We used two additional biochemical assays to characterize CisPt toxicity in cells, i.e. the WST-1 test detecting intracellular dehydrogenase activity and glutathione assay. We found considerably reduced dehydrogenase activity in HepG2 treated with ≥ 25 µM CisPt (Fig. 2C) and for all CisPt treatment of HK-2 cells in 24 h treatment (Fig. 2D), while for both cells type in 48 h treatment, the doses ≥ 5 µM CisPt had significant reduction. On the other hand, the HK-2 cell line exhibited lower susceptibility to glutathione depletion than HepG2 cells because a significant reduction of glutathione levels was found only at 100 µM CisPt (Fig. 2E,F) for 24 h treatment, while for HepG2 the significant reduction was ≥ 25 µM. For 48 h treatment, both cell types had significant reduction in similar CisPt concentrations treatments ≥ 5 µM. In conclusion, the outcomes found using the WST-1 and glutathione assays confirmed the occurrence of CisPt toxicity detected using the H33258 spectrofluorometric assay. WST-1 and glutathione assays detected cellular damage also in cells treated with 5 µM CisPt. Hence, both biochemical assays are more sensitive in detection of a cell damage in comparison to H33258 assay detecting structural nuclear changes.

**Figure 3:**
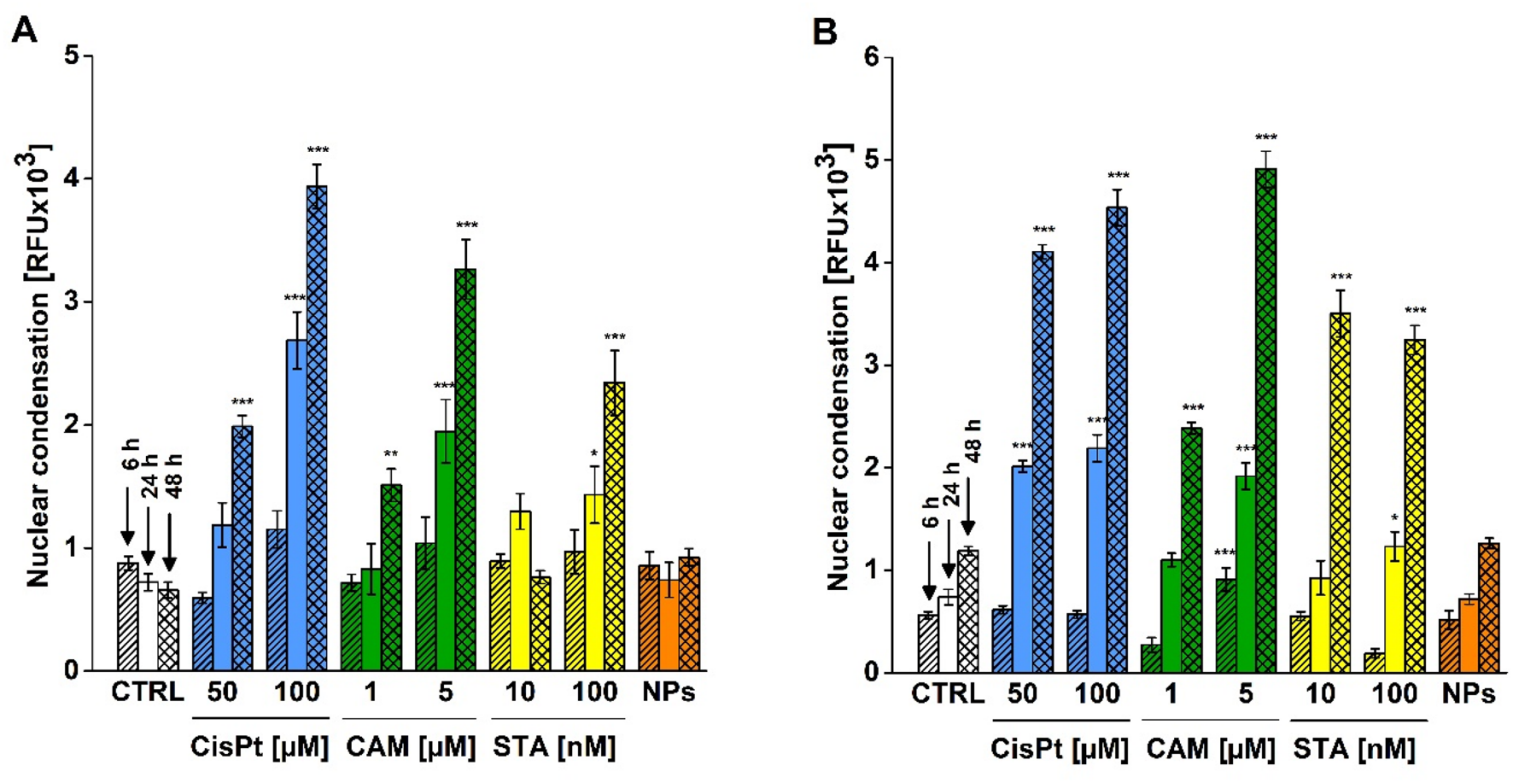
Detection of nuclear condensation and fragmentation using the H33258 spectrofluorometric assay. HepG2 (**A**) and HK-2 (**B**) cells were treated with cisplatin (CisPt, 50; 100 µM, blue columns), camptothecin (CAM, 1; 5 µM, green columns), staurosporine (STA, 10; 100 nM, yellow columns) for 6, 24 and 48 h. Control (CTRL) represents untreated cells. The cells treated with TiO_2_ P25 nanoparticles (NPs, 10 µg/mL, orange columns) were used as a negative control. After treatment, the cells were incubated with H33258 (2 µg/mL) for 5 min and intensity fluorescence (EX/EM = 352/461 nm) was measured. Then, the background fluorescence was subtracted and the extent of nuclear condensation was expressed in RFU. Data are presented as mean ± SEM (n = 8–20). (*p < 0.05; **p < 0.01; ***p < 0.001; vs. untreated cells at appropriate time interval).

In addition to CisPt, we aimed to utilize the spectrofluorometric assay for detection of nuclear changes in cells treated with other apoptotic inducers. Thus, we incubated HepG2 and HK-2 cells with camptothecin (CAM, 1; 5 µM) and staurosporine (STA, 10; 100 nM) for 6, 24 and 48 h (Fig. 3A,B). We used CisPt as a positive control and 10 µg/mL and TiO_2_ P25 nanoparticles as a negative control. All results were compared to the signal in untreated cells at appropriate time interval. Our results showed that we detected a significant increase of nuclear condensation and fragmentation in all tested compounds except of TiO_2_ P25 nanoparticles which did not induce any detected nuclear changes at all tested time durations. After 6 h of treatment, we found a significant increase of IF related to enhanced nuclear changes only in HK-2 cells treated with 5 µM CAM (*p* < 0.001). At 24 h, a significant increase of nuclear condensation and fragmentation comparing with untreated cells was detected in all HK-2 treated cells with exception of 1 µM CAM (*p* = 0.198) and 10 nM STA (*p* = 0.999*)*. We also found a significant increase of IF in HepG2 cells treated with higher concentrations of used apoptotic inducers. Thus, the H33258 assay did not detect a significant nuclear condensation and fragmentation in 1 µM CAM (*p* = 0.935), 10 nM STA (*p* = 0.135) and 50 µM CisPt (*p* = 0.382) treated HepG2 cells for 24 h.

After 48 h of treatment, we found significantly increased extent of nuclear condensation in almost all tested concentrations of compounds in both cell lines. The only exception was observed in 10 nM STA treated HepG2 cells (*p* = 0.999) which exhibited a decrease of IF in comparison with 10 nM STA treated cells for 24 h. All tested concentrations of pro-apoptotic compounds induced larger nuclear condensation after 48 h incubation in comparison to 24 h and the cells treated with higher concentrations of tested compounds exhibited larger nuclear impairment in comparison to lower dose. The highest IF signal was detected in HepG2 and HK-2 cells treated with 100 µM CisPt and 5 µM CAM at 48 h. In conclusion, our results confirmed that the H33258 spectrofluorometric assay was capable to detect increased nuclear condensation and fragmentation in all tested apoptotic inducers relatively to concentration and incubation period (Fig. 3A,B).

### Comparison of H33258 spectrofluorometric assay with apoptosis detecting methods

To evaluate the outcomes of newly developed H33258 spectrofluorometric assay, we aimed to compare the results with other methods characterizing pro-apoptotic processes in cells. Therefore, we treated HepG2 and HK-2 cells with CisPt (50 and 100 µM) for 6, 24 and 48 h and characterized nuclear changes and pro-apoptotic activation using four additional measurements, i.e. caspase 3/7 activity, PARP-1 and JNK proteins expression, TUNEL assay and DNA ladder.

**Figure 4:**
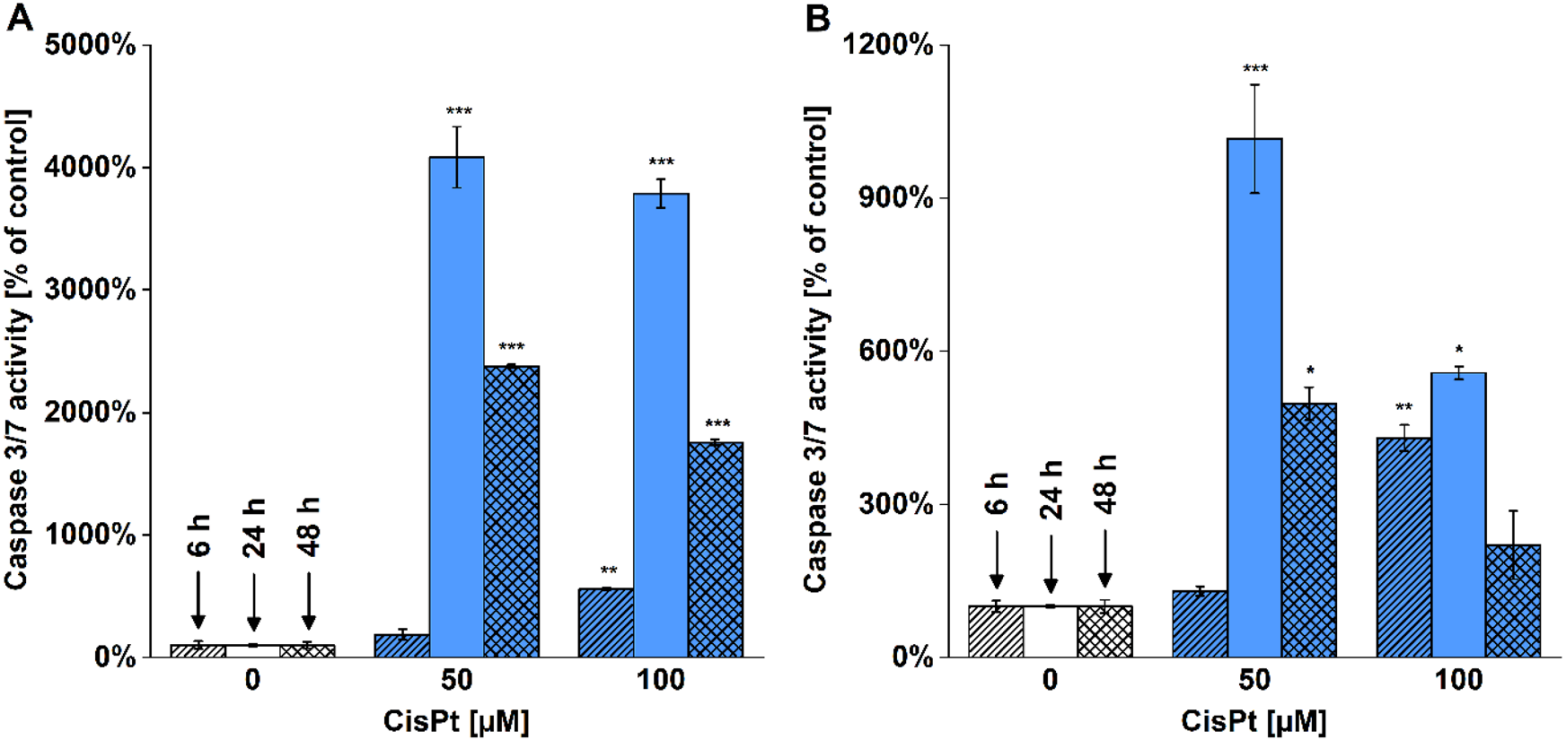
Caspase 3/7 activity in CisPt treated cells. HepG2 (**A**) and HK-2 (**B**) cells were treated with CisPt (50 and 100 µM) for 6, 24 and 48 h. After incubation, the activity of caspases 3/7 was measured (EX/EM = 485/535 nm). Data are presented as mean ± SEM (n = 2). (*p < 0.05; **p < 0.01; ***p < 0.001; vs. untreated cells at appropriate time interval).

We measured the activity of caspases 3 and 7 to evaluate apoptotic activation in the cells. Despite detecting a significant increase in caspase 3/7 activity in both cell lines treated with 100 µM CisPt for 6 h, the largest enhancement of caspase 3/7 activity was detected in cells treated with 50 µM CisPt for 24 h (Fig. 4). At 48 h, the caspase 3/7 activities remained increased although we found a decrease in comparison to cells treated for 24 h.

Then, we analyzed the levels of PARP-1, PARP-1 fragment (fPARP-1), pJNK and β-actin. PARP-1 protein serves as a substrate for caspase 3 during apoptosis. Thus, PARP-1 cleavage leading to fPARP-1 production is related to the extent of caspase 3 activation. After 24 h of treatment, we detected PARP-1 fragmentation in cells treated with both concentrations of cisplatin which persisted until 48 h only in HepG2 cells (Table 1). Phosphorylation of JNK1 and JNK2 can also correspond with activated apoptotic process and that is why we aimed to estimate their levels. We observed that pJNK1 levels were increased in all tested time incubation with CisPt in HepG2 cells. In HK-2 cells, protein expressions of pJNK1 and pJNK2 were stimulated predominantly at 24 h of treatment. Thus, the detected increase of pJNK levels correlated strongly with PARP fragmentation in both cell lines.

**Table 1:** PARP and JNK protein expressions in CisPt treated cells.

Time (h)	CisPt (µM)	HepG2				HK-2			
		PARP-1	fPARP-1	pJNK1	pJNK2	PARP-1	fPARP-1	pJNK1	pJNK2
6	0	2.3	0	0	0	0.4	0.1	0	0
	50	2.3	0	0.2	0.2	0.9	0.1	0	0
	100	1.8	0	0.4	0.3	0.7	0.5	0.1	0.1
24	0	1.9	0	0	0	1.6	0.1	0.1	0
	50	1.9	1.3	0.2	0.2	0.9	1.5	0.2	0.1
	100	2.7	2.0	0.7	0.6	0.8	1.5	0.3	0.2
48	0	3.0	0	0	0	0.9	0	0	0
	50	3.2	1.3	0.5	0	0.9	0.1	0	0
	100	3.2	1.0	1.2	1.0	0	0	0	0

HepG2 and HK-2 cells were treated with CisPt (50 and 100 µM) for 6, 24 and 48 h. After incubation, the protein expression of PARP-1, fPARP-1, pJNK1 and pJNK2 was analyzed using capillary Western immunoassay. The results were expressed as a ratio: $\frac{{{\text{area}}\,{\text{of}}\,{\text{the}}\,{\text{peak}}\,{\text{of}}\,{\text{interest}}}}{{{\text{area}}\,{\text{of}}\,{\text{the}}\,{\text{peak}}\,{\text{of}}\,\upbeta {\text{ - actin}}}}$.

**Figure 5:**
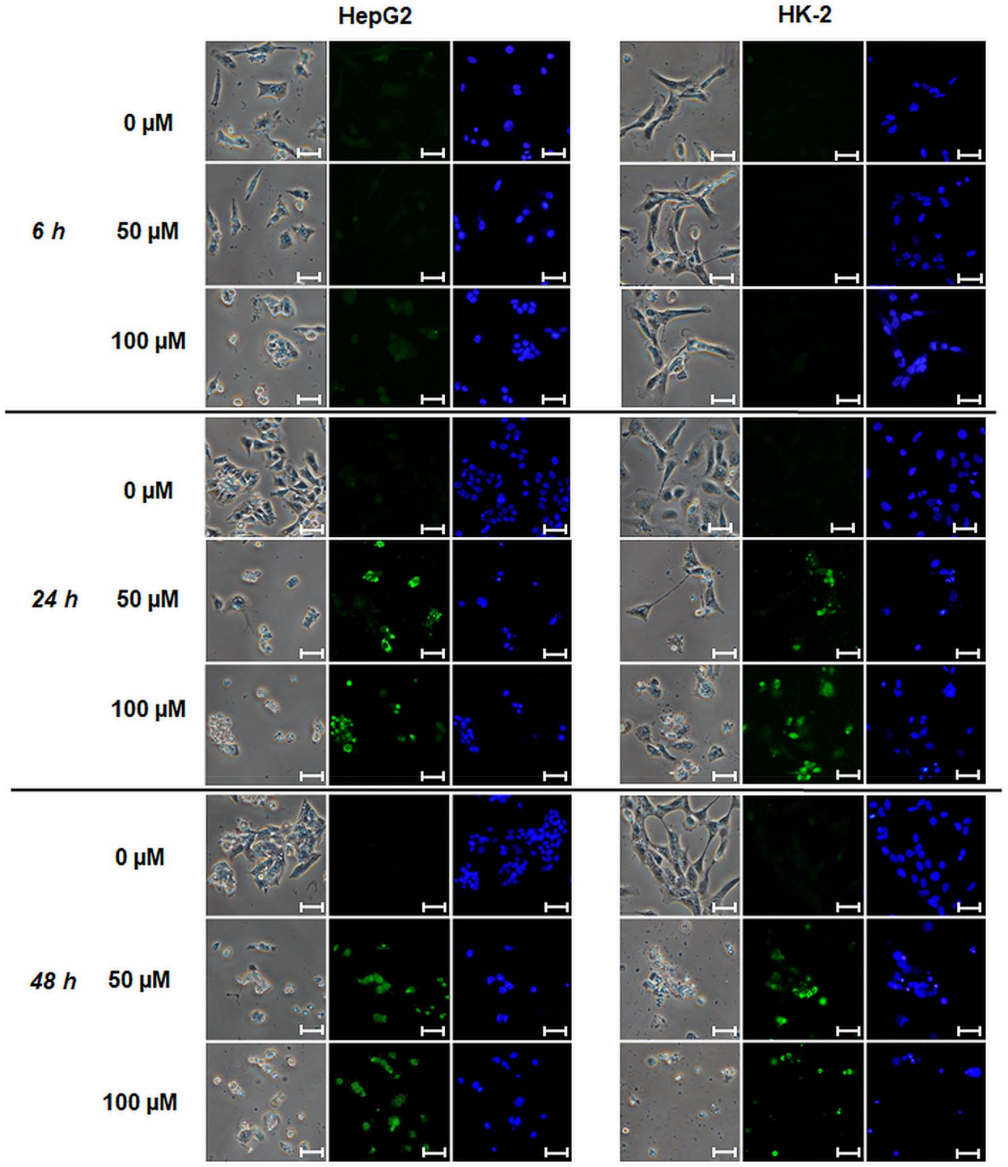
TUNEL assay in CisPt treated cells. HepG2 and HK-2 cells were treated with CisPt (50 and 100 µM) for 6, 24 and 48 h. Then, the cells and nuclei were visualized using phase contrast (left, magnification 400 ×), TUNEL (FITC 480/30 nm; middle) and Hoechst 33258 stainings (DAPI 375/28 nm; right). Scales correspond to 25 µm.

TUNEL assay was used for detection of DNA strand breaks in CisPt treated cells (Fig. 5). After 6 h of treatment, a weak fluorescence demonstrating presence of DNA strand breaks was observed only in 100 µM CisPt treated HepG2 cells. At longer time durations, TUNEL staining detected strong DNA fragmentation in both cell lines treated with both 50 and 100 µM CisPt (Fig. 5).

**Figure 6:**
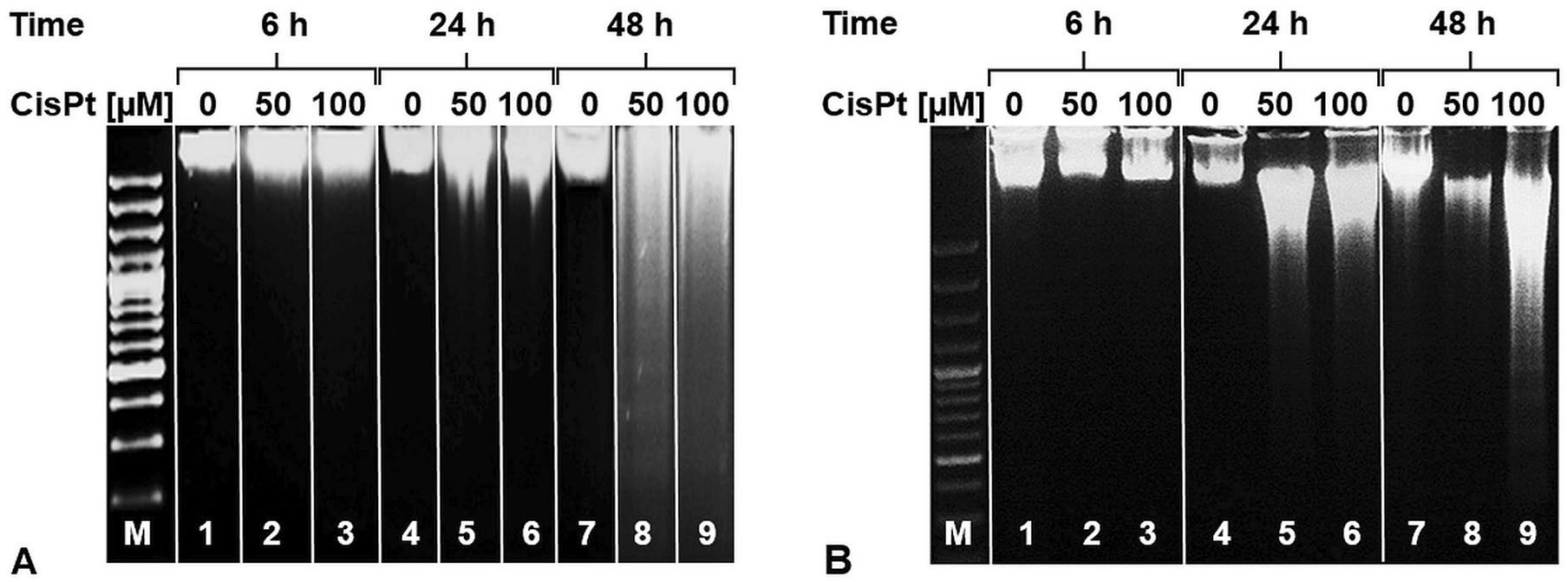
DNA ladder assay in CisPt treated cells. HepG2 (**A**) and HK-2 (**B**) cells were treated with CisPt (50 and 100 µM) for 6, 24 and 48 h. After DNA isolation, DNA ladder assay was performed. (0, untreated cells; 50, 50 µM CisPt; 100, 100 µM CisPt; M, 100 bp marker). Original gels images were included into the Supplementary file.

At final, we used DNA ladder assay in CisPt treated cells to estimate the extent of DNA fragmentation. The internucleosomal DNA fragmentation can occur as the terminal feature of apoptosis. Thus, we detected DNA ladder pattern only in CisPt treated HepG2 cells after 48 h (Fig. 6A) and in HK-2 cells after 24 and 48 h (Fig. 6B).

We summarized and compared the results obtained using all methods detecting apoptosis in CisPt treated HepG2 and HK-2 cells (Table 2). We proved that the initial increase in caspase 3/7 activity was followed by fragmentation of PARP-1, DNA fragmentation detected using TUNEL assay and formation of DNA ladder. In addition, Table 2 shows that the newly developed spectrofluorometric assay using the H33258 dye provided outcomes fully comparable with TUNEL assay which has been used as a standard method detecting increased levels of DNA fragmentation in cells.

**Table 2:** Comparison of assays detecting pro-apoptotic changes.

	H33258	Caspases	TUNEL	fPARP-1	DNA ladder
**6 h**					
HepG2	+	+	+/−	−	−
HK-2	−	++	−	+	−
**24 h**					
HepG2	++	++	++	++	−
HK-2	++	++	++	++	++
**48 h**					
HepG2	++	++	++	++	++
HK-2	++	−	++	−	++

HepG2 and HK-2 cells were treated with 100 µM CisPt for 6, 24 and 48 h. After treatment, the nuclear changes were detected using five methods, i.e. developed spectrofluorometric assay using Hoechst 33258 (= H33258), caspase 3/7 activity, TUNEL assay, protein expression of PARP-1 fragment (fPARP-1) and DNA ladder. (−, negative; +/−, moderately positive; +, positive; ++, strongly positive).

## Discussion

DNA staining using Hoechst fluorescent probes belongs to commonly used approaches for visual detection of nuclear condensation. Recent scientific studies have described the use of H33258 for fluorescence detection of cell shrinkage, chromatin condensation, nuclear fragmentation and apoptotic bodies formation in cells^16,28–32^. Surprisingly, these reports used H33258 only for microscopic detection of nuclear changes. Thus, we aimed to develop a quantitative spectrofluorometric method for the detection of nuclear condensation and fragmentation using H33258 in cultured cells.

In this study, we used HepG2 and HK-2 cells. Doubling times of HepG2 and HK-2 cells are ca 48 h and 54 h, respectively^36,37^. Thus, the influence of cell cycle duration on obtained results was rather negligible. Firstly, we optimized H33258 concentration which is of crucial importance due to potential toxicity of the dye. Some studies reported that H33258 levels higher than 5 µg/mL are toxic to cells especially after long-term exposure^38,39^. H33258 concentration used in studies has been 1–5 µg/mL H33258 in fluorescence microscopy^40,41^ and 0.5–2 µg/mL H33258 in flow cytometry^21,42,43^. Therefore, we decided to estimate H33258 concentrations in the range of 0.1–5 µg/mL for development of the spectrofluorometric assay. We selected 2 µg/mL H33258 to be optimal for treatment of cells without risk of cell impairment during the spectrofluorometric measurement for 5 min.

After introduction of the spectrofluorometric assay´s conditions, we aimed to utilize the assay for detection of chromatin condensation and nuclear fragmentation in two cell lines of different origin, i.e. human liver carcinoma HepG2 cells and proximal tubular epithelial HK-2 cells, treated with three inducers of apoptosis (cisplatin, camptothecin, staurosporine). Cisplatin is an anticancer drug capable to induce apoptotic DNA fragmentation in cells^44,45^. CisPt interracts with adenine or guanine forming adducts which inhibit DNA replication and transcription leading to p53 or MAPK activation^46^. Camptothecin is an inhibitor of DNA topoisomerase I^47–49^ causing formation of DNA strand breaks and p53 activation. Staurosporine is a strong inhibitor of protein kinases inducing DNA fragmentation^50,51^. Moreover, the cells were incubated with TiO_2_ P25 nanoparticles which have been reported to possess no capability to induce nuclear condensation or DNA fragmentation in HepG2 cells^52^.

According to our results, we showed that the developed spectrofluorometric assay using H33258 was capable to detect nuclear changes. In addition, the intensity of fluorescence reflected different extent of nuclear condensation and fragmentation in comparison of 6, 24 and 48 h of incubation in CAM, STA and CisPt treated cells. Our finding of nuclear condensation and fragmentation detected using the new spectrofluorometric method after treatment with 5 µM CAM for 6 h can be supported by a study of Rath et al.^48^ who found presence of apoptotic nuclei in 5 µM CAM treated thyroid carcinoma cells after 8 h of treatment using TUNEL assay. Another study focused on estimation of CAM-dependent induction of apoptosis showed occurrence of apoptotic changes in 1 µM CAM treated HepG2 cells after 24 h^53^ using flow cytometry. Therefore, the newly developed H33258 spectrofluorometric method provided similar outcomes to other scientific reports studying apoptosis induced by camptothecin.

To characterize staurosporine effect in cells, we used the concentrations which have been reported to be sufficient to induce the cell death^50,54^. Our results on detection of changes in nuclear structure using the spectrofluorometric method are consistent with a study of Ding et al.^50^ who also detected DNA fragmentation in 10 nM STA treated HepG2 cells after 24 h of treatment using TUNEL assay. Another paper of Deshmuk and Johnson^51^ described a finding of increased DNA fragmentation in 100 nM STA treated sympathetic neurons after 24 h using TUNEL staining as well. Therefore, we can conclude again that the spectrofluorometric method using H33258 provided results similar to the outcomes of other studies characterizing staurosporine action in cells.

Finally, we aimed to use the spectrofluorometric assay using the H33258 dye for characterization of cisplatin action in cells and to compare obtained results with the outcomes of other apoptosis detecting assays. Occurrence of significant pro-apoptotic nuclear changes in cisplatin-treated cells has been broadly reported after 24 h of incubation. A study of Yang et al.^45^ detected increased DNA fragmentation in 50 µM CisPt treated HK-2 cells for 24 h using TUNEL assay. Similar results were obtained also in CisPt treated LLC-PK1 kidney cells using TUNEL assay^55^ and in HepG2 cells using fluorescence microscopy and flow cytometry^44^. Based on these papers, our findings of increased nuclear condensation and fragmentation in 50 and 100 µM CisPt treated HepG2 and HK-2 cells using the developed spectrofluorometric assay are fully consistent with the literature.

The latter goal of our study was to compare the outcomes obtained using the developed spectrofluorometric assay in CisPt treated cells with outcomes of other, frequently used assays detecting apoptosis, i.e. caspase 3/7 activity, JNK/PARP proteins expression, TUNEL assay and DNA ladder. Caspase 3/7 activation detected in our experiments after 6 h of treament was also described in a paper of Schweyer et al.^56^ in 50 µM CisPt treated NCCIT cells. Persisting activation of caspases 3/7 was reported in a number of studies, e.g. in UBOC1, HK-2 and SH-SY5Y cells after treatment with 5,10 a 20 µM CisPt for 24 h^57^ and in HepG2 cells treated with 25 µM CisPt for 12, 24 and 48 h^58^.

Capillary Western immunoassay was performed for detection of protein expression changes of PARP-1, JNK1 and JNK2. PARP-1 reflects the extent of DNA damage^59^ because it is cleaved by caspases 3/7 to two specific fragments of 24 and 89 kDa^60^. Thus, cleavage of PARP-1 protein by caspases is usually mentioned to be a hallmark of apoptosis^61^. Our results on detecting PARP-1 cleavage after treatment with 50 µM CisPt for 24 h are similar to the study of Ju et al.^62^ who detected the PARP-1 cleavage in HK-2 cells treated with 40 µM CisPt for 24 h. JNKs were reported to be strongly activated via phosphorylation in cells incubated with cisplatin^63^. In our study, expression of phosphorylated JNK isoforms (JNK1 and JNK2) was elevated after cisplatin treatment in all time intervals in dose-dependent manner compared to control cells which is similar to reported finding on increased pJNK expression in HeLa cells after incubation with 50 and 100 µM CisPt for 6 h^64^, and in pig kidney epithelial cells after 25 µM CisPt treatment for 24 h^65^.

Finally, TUNEL and DNA ladder assays were performed in CisPt treated cells. Both assays belong among frequently used methods for detection of DNA strand breaks and fragmentation^14,45^.

We found that TUNEL assay detected DNA fragmentation after 24 h of CisPt treatment. Thus, we obtained similar results to finding on DNA fragmentation detected using TUNEL assay in 50 µM CisPt treated HK-2 cells^45^ and LLC-PK1 cells^55^ after 24 h. The detection of internucleosomal DNA fragments using DNA ladder assay is in accordance with outcomes of studies of Qin et al. (2002) and Lau^66^ reporting occurrence of DNA ladder in CisPt treated HepG2 and LLC-PK1 cells after 24 and 48 h. We summarize that the findings of DNA fragmentation and nuclear condensation detected using all the biochemical assays in HepG2 and HK-2 cells after cisplatin treatment presented in this study are in accordance to published reports of other authors testing cisplatin as well.

## Conclusion

We developed a spectrofluorometric method using Hoechst 33258 staining which detects nuclear condensation and fragmentation in intact cells. Our results showed that the spectrofluorometric method was capable to detect the nuclear changes in three typical pro-apoptotic agents, i.e. cisplatin, camptothecin, staurosporine, and in two cell lines of different origin. In addition, we compared the results obtained using the spectrofluorometric assay with outcomes of other methods characterizing apoptotic processes. Based on this comparison, we conclude that here developed spectrofluorometric method is capable to detect nuclear pro-apoptotic changes of similar sensitivity and specificity to that of caspase 3/7 activity measurement and TUNEL assay. Therefore, we suppose that the spectrofluorimetric H33258 method could join other routinely used methods characterizing apoptosis in cells. In comparison to TUNEL assay, the developed spectrofluorometric assay possesses several advantages (e.g. rapid processing, quantitative evaluation, low-cost) implying its potential use in assessing nuclear condensation and fragmentation in routine laboratory practice and in high-throughput screening studies.

## Materials and methods

### Chemicals

Hoechst 33258 solution (H33258, 1 mg/mL) and all other chemicals (formaldehyde, Triton X-100, BSA, NaCl, NaOH, Tris base, Na_2_EDTA·2H_2_O, PBS 10 ×, cisplatin, staurosporine, camptothecin, TiO_2_ P25) were purchased from Sigma Aldrich, USA.

### Cell culture and treatment

All materials for cell culture were purchased from Sigma-Aldrich (USA) if not otherwise specified. HepG2 cells (ATCC HB-8065), a human hepatocellular carcinoma cell line, were purchased from ATCC (Manassas, VA, USA). HepG2 cells were cultured in Dulbecco's Modified Eagle's Medium with high glucose content (4500 g/L, w/wo phenol red) supplemented with 10% (v/v) fetal bovine serum, 50 μg/mL penicillin, 50 μg/mL streptomycin, 10 mM HEPES and 2 mM glutamine, maintained at 37 °C in a sterile, humidified atmosphere of 5% CO_2_. All the experiments were conducted using HepG2 cells in passages 4–15.

Human kidney, HK-2 cells (ATCC CRL-2190), a proximal tubular epithelial cell line derived from normal adult human kidney cells immortalized by transduction with human papillomavirus (HPV 16) DNA fragment^67^, were purchased from the ATCC (Manassas, VA, USA). The cells were cultured in supplemented Dulbecco’s modified Eagle’s medium (DMEM/F12 = 1:1) with 5% (v/v) fetal bovine serum, 1 mM pyruvate, 10 µg/mL insulin, 5.5 µg/mL transferrin, 5 ng/mL sodium selenite, 50 µg/mL penicillin, 50 µg/mL streptomycin, and 5 ng/mL epidermal growth factor according to a published protocol^68,69^. All the experiments were conducted using the HK-2 cells in passages 5–15.

HepG2 and HK-2 cells were tested for mycoplasma contamination using the MycoAlert Mycoplasma Detection Kit (Lonza). All cells used in the experiments were mycoplasma free. Short tandem repeat (STR) analysis (i.e., DNA fingerprinting) was used for HepG2 and HK-2 cell line authentication using a commercial kit in Generi Biotech (Czech Republic). The STR analysis proved 100% conformity of both HepG2 and HK-2 cells with the reference standards.

The HepG2 and HK-2 cells were seeded in 100 µL of appropriate cell culture medium in 96-well plates at density of 1.5 × 10^4^ and 2 × 10^4^ cells per well for 24 h (if not stated otherwise). To induce cell impairment, we used: cisplatin (CisPt, 0–100 µM), camptothecin (CAM; 0–5 µM), staurosporine (STA; 0–100 nM) and TiO_2_ P25 nanoparticles (NPs; 0–10 µg/mL). All compounds were diluted in appropriate cell culture mediums to obtain final concentrations. After seeding, the culture medium was replaced by 100 µL of medium containing a tested compound and the cells were treated for 6, 24 and 48 h. To characterize the extent of cell impairment, we used the newly developed spectrofluorometric assay using H33258 together with caspase activity and proteins expression measurements, TUNEL and DNA ladder assays.

### Hoechst 33258 spectrofluorometric assay

To develop a spectrofluorometric method for detection of changes in nuclear condensation and fragmentation in intact cells, we used a fluorescence dye H33258. To optimize the assay, we used HepG2 and HK-2 cells at confluency 50–70%. After treatment with tested compounds (CisPt, CAM, STA, NPs) for 6, 24 and 48 h, the cells grown in a 96-well plate were centrifuged (5 min, 8000*g*) at RT. Then, 70 µL of a supernatant was replaced with 70 µL of warmed phosphate-buffered saline (PBS 1 ×, 37 °C) and 10 µL of H33258 solution (in PBS 1 ×) was added to a well. The final concentrations of H33258 in a well were 0.1–5 µg/mL. Then, the cells were incubated with H33258 for 60 min during optimization of the assay, or for 5 min at optimal conditions and the spectrofluorometric measurement was performed at EX/EM = 352/461 nm (EX/EM slit widths 25/25 nm) using a Tecan Spark fluorescence microplate reader (Tecan, Switzerland) while incubated at 37 °C. The samples were measured at least in triplicates. After background subtraction, the fluorescence signal was presented in Relative Fluorescence Units (RFU) as mean ± SEM. All spectrofluorometric measurements presented here were repeated at least in three independent experiments.

### Dehydrogenase activity measurement

Dehydrogenase activity was evaluated by WST-1 test (Roche, Germany). The WST-1 test measures the activity of intra- and extramitochondrial dehydrogenases. After cell treatment, 10 µL of WST-1 reagent was added to the treated cells. The absorbance change (0–1 h) was measured spectrophotometrically at wavelength of 440 nm using a Tecan Spark fluorescence microplate reader (Tecan, Switzerland). The cell viability was expressed as the percentage of intra- and extramitochondrial dehydrogenases activity relative to that in control cells (= 100%). The results were expressed as mean ± SD.

### Glutathione assay

Glutathione levels were measured using the monochlorobimane spectrofluorometric assay^70^. After cell treatment, 20 µL of the bimane solution was added to cells to obtain the final concentration 40 µM and spectrofluorometric measurement was started immediately. The fluorescence increase (EX/EM = 394/490 nm) was measured for 10 min using a Tecan Spark fluorescence microplate reader (Tecan, Switzerland). The fluorescence was expressed as the slope of change in fluorescence over time. The glutathione levels were expressed as the percentage relative to the glutathione levels in control cells (= 100%). The results were expressed as mean ± SD.

### Caspase 3/7 activity measurement

Caspase 3/7 activity in HepG2 and HK-2 cells was detected using ApoONE Homogeneous Caspase-3/7 Assay (Promega, USA) according to the manufacturer´s instructions. Briefly, 100 µL of the caspase 3/7 working solution was added to treated cells. After mixing, the cells were incubated for 30 min. Then, the fluorescence (EX/EM = 485/535 nm) was measured in duplicates using a Tecan Spark fluorescence microplate reader (Tecan, Switzerland) while incubated at 37 °C. The caspase 3/7 activity levels were expressed as the percentage relative to the caspase 3/7 activity levels in control cells (= 100%). The results were expressed as mean ± SEM.

### Capillary Western Immunoassay

Capillary Western Immunoassay was performed in protein lysates from HepG2 and HK-2 cells cultured in 6-well plates at density of 5 × 10^5^ and 1.3 × 10^6^ cells per well, respectively. After seeding, the culture medium was replaced by 2 mL of CisPt solution and the cells were treated for 6, 24 and 48 h. After treatment, the cells were washed twice with PBS 1 × and protein lysates were prepared by lysing cells with 400 µL of RIPA buffer (Sigma-Aldrich, USA) with MS-safe protease and phosphatase inhibitor (Sigma-Aldrich, USA) on ice. Capillary Western Immunoassay was performed according to manufacturer´s instructions (Protein Simple, USA). Briefly, protein lysates were analyzed on a Wes system (ProteinSimple, USA) using a 12–230 kDa Separation Module (Biotechne, UK). Levels of phosphorylated JNK (pJNK, primary antibody 1:50, Promega, USA), poly-(ADP-ribose) polymerase-1 (PARP-1; primary antibody 1:100; Cell Signalling, USA) were normalized using the reference protein β-actin (primary antibody 1:500, Sigma-Aldrich, USA). The peaks were analyzed using Compass software (Protein Simple, USA). Two criteria were used for the discrimination of signals from the background: (1) the peak high must be higher or equal to 1000 and (2) the peak’s signal-to-noise ratio given by the software must be higher or equal to 10. The results were counted as:$\frac{{{\text{area}}\,{\text{of}}\,{\text{the}}\,{\text{peak}}\,{\text{of}}\,{\text{interest}}}}{{{\text{area}}\,{\text{of}}\,{\text{the}}\,{\text{peak}}\,{\text{of}}\,\upbeta {\text{ - actin}}}}$.

### TUNEL assay

TUNEL assay followed by H33258 staining of nuclei was performed in HepG2 and HK-2 cells cultured in 200 µL of appropriate cell culture medium on cell culture chamber slides at density of 1.5 × 10^5^ and 2 × 10^5^ cells per well, respectively. After seeding, the culture medium was replaced by 200 µL of CisPt solutions and the cells were treated for 6, 24 and 48 h. TUNEL assay was performed using Click-iT TUNEL Alexa Fluor 488 Imaging Assay kit (ThermoFisher Scientific, USA) according to manufacturer´s instructions. The cells were fixed with 12% formaldehyde for 15 min at 37 °C. Then, the cells were permeabilized with 0.2% Triton X-100 for 15 min at 37 °C, washed with PBS 1 × and incubated with terminal deoxynucleotidyl transferase (TdT) buffer for 10 min at 37 °C. After incubation, cells were mixed with a TdT reaction mixture (TdT buffer, 5-Ethynyl-2′-deoxyuridine 5′-triphosphate, TdT) and incubated for 1 h at 37 °C. Then, the cells were washed with 3% bovine serum albumin (BSA) and Click IT reagent for fluorescent staining was added for 30 min at 37 °C. After PBS 1 × washing, H33258 at a final concentration of 2 µg/mL was used to visualize the cell nuclei. DNA strand breaks (FITC filter, 480/30 nm) and cell nuclei (DAPI filter, 375/28 nm) were visualized with an Eclipse 80i fluorescence microscope (Nikon, Japan).

### DNA ladder

DNA ladder was performed in HepG2 and HK-2 cells cultured in 6-well plates at density of 5 × 10^5^ and 1 × 10^6^ cells per well, respectively. After seeding, the culture medium was replaced by 2 mL of CisPt and the cells were treated for 6, 24 and 48 h. DNA was isolated from treated cells using The ApoTarget Quick Apoptotic DNA Ladder Detection Kit (Invitrogen, USA). Isolated DNA samples were loaded onto a 1.5% agarose gel with 0.5 mg/mL ethidium bromide (Top-Bio, Czech Republic) followed by electrophoresis (5 V/cm). Finally, DNA was visualized by an ultraviolet gel documentation system (Vilber Lourmat, Germany) at wavelength 254 nm. GeneRuler 100 bp DNA ladder (ThermoFisher Scientific, USA) was used as a DNA size standard.

### Statistical analysis

Statistical analysis was performed using OriginPro 9.0.0 (OriginLab, USA). Statistical significance was analyzed after normality testing using one-way analysis of variance (ANOVA) followed by Tukey’s test at significance level *p* = 0.05 (*p < 0.05; **p < 0.01; ***p < 0.001).

## Supplementary Information

Supplementary Figures.
