# Author Correction: Quantitative spectrofluorometric assay detecting nuclear condensation and fragmentation in intact cells

**DOI:** 10.1038/s41598-021-97792-5

**Published:** 2021-09-03

**Authors:** Pavlina Majtnerova, Jan Capek, Filip Petira, Jiri Handl, Tomas Rousar

**Affiliations:** Department of Biological and Biochemical Sciences, Faculty of Chemical Technology, University of Pardubice, Studentska 573, 532 10 Pardubice, Czech Republic

Correction to: *Scientific Reports* 10.1038/s41598-021-91380-3, Published online 07 June 2021

The original version of this Article contained an error in the spelling of the authors Pavlina Majtnerova, Jan Capek, Filip Petira, Jiri Handl and Tomas Rousar which were incorrectly given as Majtnerova Pavlina, Capek Jan, Petira Filip, Handl Jiri and Rousar Tomas respectively.

The original Article and accompanying Supplementary Information files have been corrected.

