# Supplementary material for: Quantitative spectrofluorometric assay detecting nuclear condensation and fragmentation in intact cells
Source: Sci Rep. 2021 Jun 7;11:11921. doi: 10.1038/s41598-021-91380-3 (PMC8184882; doi:10.1038/s41598-021-91380-3)
Supplement: Supplementary file 1 — Supplementary Figures. [file 41598_2021_91380_MOESM1_ESM.docx]

**SUPPLEMENTARY INFORMATION**

**Manuscript title: Quantitative spectrofluorometric assay detecting nuclear condensation and fragmentation in intact cells**

**Pavlina Majtnerova, Jan Capek, Filip Petira, Jiri Handl, Tomas Rousar ^*^**

*Affiliation: Department of Biological and Biochemical Sciences, Faculty of Chemical Technology, University of Pardubice, Studentska 573, 532 10 Pardubice, Czech Republic.*

**Supplementary Figures**

Supplementary Figure S1. Original gel image of DNA ladder assay in CisPt treated HepG2 cells for 6 h.

Supplementary Figure S2. Original gel image of DNA ladder assay in CisPt treated HepG2 cells for 24 h.

Supplementary Figure S3. Original gel image of DNA ladder assay in CisPt treated HepG2 cells for 48 h.

Supplementary Figure S4. Original gel image of DNA ladder assay in CisPt treated HK-2 cells for 6, 24 and 48 h.

**
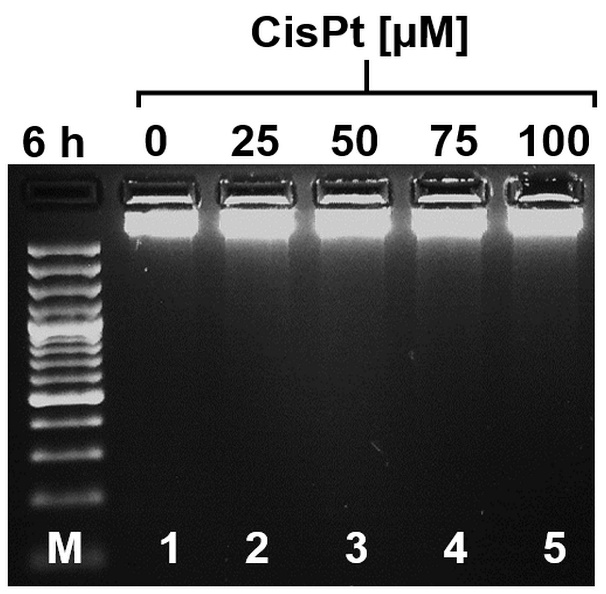
Supplementary Figures**

**Figure S1. Original gel image of DNA ladder assay in CisPt treated HepG2 cells for 6 h.** *M*, GeneRuler 100 bp DNA ladder; *1-5*, HepG2 cells treated with CisPt (0; 25; 50; 75; 100 µM) for 6 h. In Fig. 6, only samples from 0, 50 and 100 µM CisPt treated cells were presented.

**
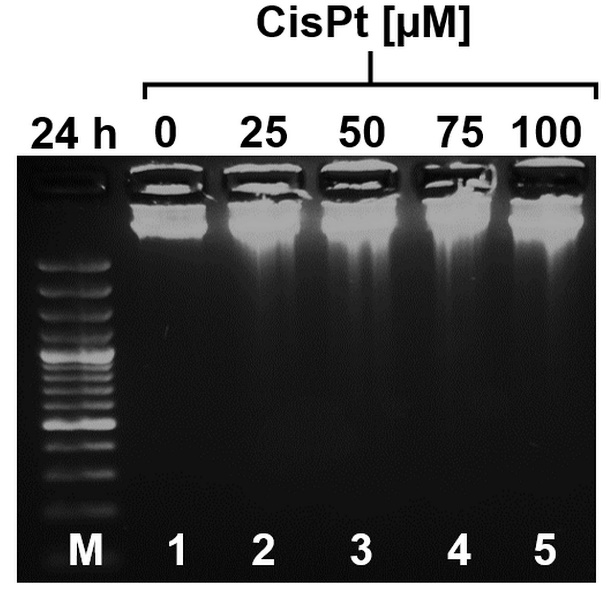
**

**Figure S2. Original gel image of DNA ladder assay in CisPt treated HepG2 cells for 24 h.** *M*, GeneRuler 100 bp DNA ladder; *1-5*, HepG2 cells treated with CisPt (0; 25; 50; 75; 100 µM) for 6 h. In Fig. 6, only samples from 0, 50 and 100 µM CisPt treated cells were presented.

**
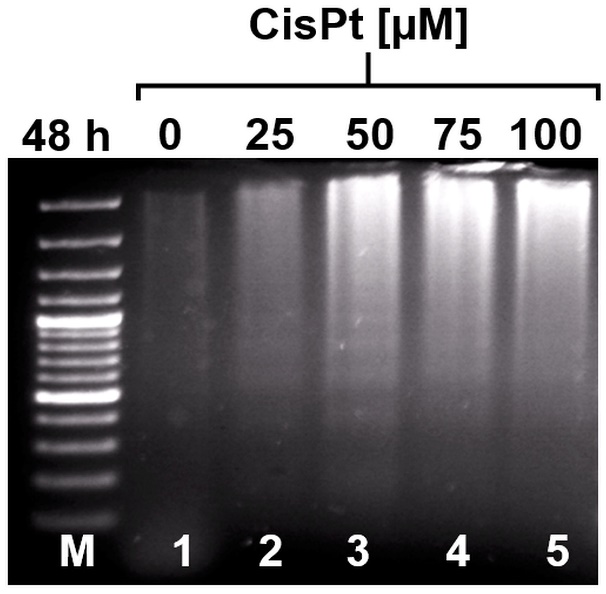
**

**Figure S3. Original gel image of DNA ladder assay in CisPt treated HepG2 cells for 48 h.** *M*, GeneRuler 100 bp DNA ladder; *1-5*, HepG2 cells treated with CisPt (0; 25; 50; 75; 100 µM) for 48 h. In Fig. 6, only samples from 0, 50 and 100 µM CisPt treated cells were presented.


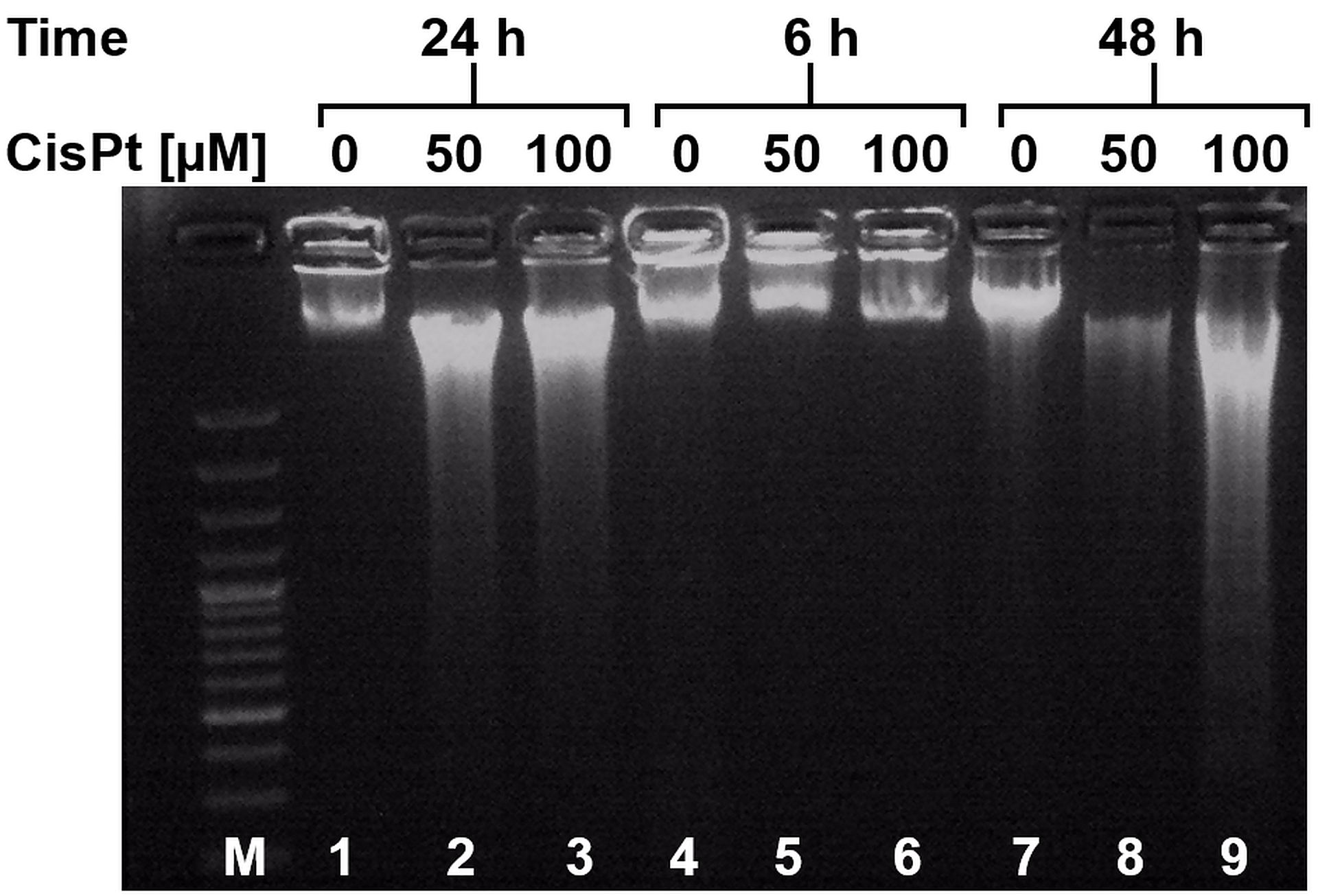


**Figure S4. Original gel image of DNA ladder assay in CisPt treated HK-2 cells for 6, 24 and 48 h.** *M*, GeneRuler 100 bp DNA ladder; *1-9*, HK-2 cells treated with CisPt (0; 50; 100 µM) for 6, 24 and 48 h. In Fig. 6, the columns were rearranged according to increasing time duration.
